# Supplementary material for: Improved Genetic Characterization of Hypercholesterolemia in Latvian Patients with Familial Hypercholesterolemia: A Combined Monogenic and Polygenic Approach Using Whole-Genome Sequencing
Source: Int J Mol Sci. 2024 Dec 16;25(24):13466. doi: 10.3390/ijms252413466 (PMC11677843; doi:10.3390/ijms252413466)
Supplement: Supplementary file 1 [file ijms-25-13466-s001.zip › ijms-3363591-supplementary.pdf]

Table S1. Patient characteristics in non-FH subgroups.

Characteristic	High LDL-C PRS	High LPA PRS	High LDL-C and LPA PRS	Priority VUS, both PRS <P_{95}	Genetically unexplained
**Number of cases**	n=45	n=16	n=7	n=12	n=179
**Male, n (%)**	19 (42.2%)	5 (31.3%)	2 (28.6%)	6 (50.0%)	72 (40.2%)
**Age**					
**Mean, SD**	52.2±10.8	53.1±9.6	51.6±13.2	45.9±14.2	53.8±11.4
**Median, IQR**	53.0 [44.5-59.0]	54 [45.5-60.8]	50.0 [38.0-66.0]	41.5 [34.3-56.5]	54.0 [47.0-62.0]
**Highest documented LDL-C (mmol/L)**					
**Mean (SD**	6.7±0.9	6.9±0.7	6.2±0.9	7.1±1.0	6.8±1.3
**Median (IQR)**	6.7 [5.9-7.3]	6.8 [6.5-7.0]	6.5 [5.2-7.0]	7.0 [6.4-7.8]	6.7 [6.0-7.4]
**DLCN criteria***					
**Median (IQR)**	6.0 [5.0-6.5]	7.0 [6.0-10.3]	6.0 [5.0-8.0]	6.0 [5.3-9.3]	6.0 [5.0-7.0]
**Tendon xanthomas, n (%)**	6 (13.3%)	3 (18.8%)	0 (0%)	2 (16%)	25 (14.0%)
**Premature arcus cornealis <45 years**	1 (2.2%)	1 (6.3%)	0 (0%)	0 (0%)	3 (1.7%)

FH – familial hypercholesterolemia; P_{95} – 95th percentile, DLCN - Dutch Lipid Clinic Network, LDL-C - low-density lipoprotein cholesterol, LPA - lipoprotein(a), SD - standard deviation, IQR - interquartile range; VUS - variant of uncertain significance, PRS - polygenic risk score

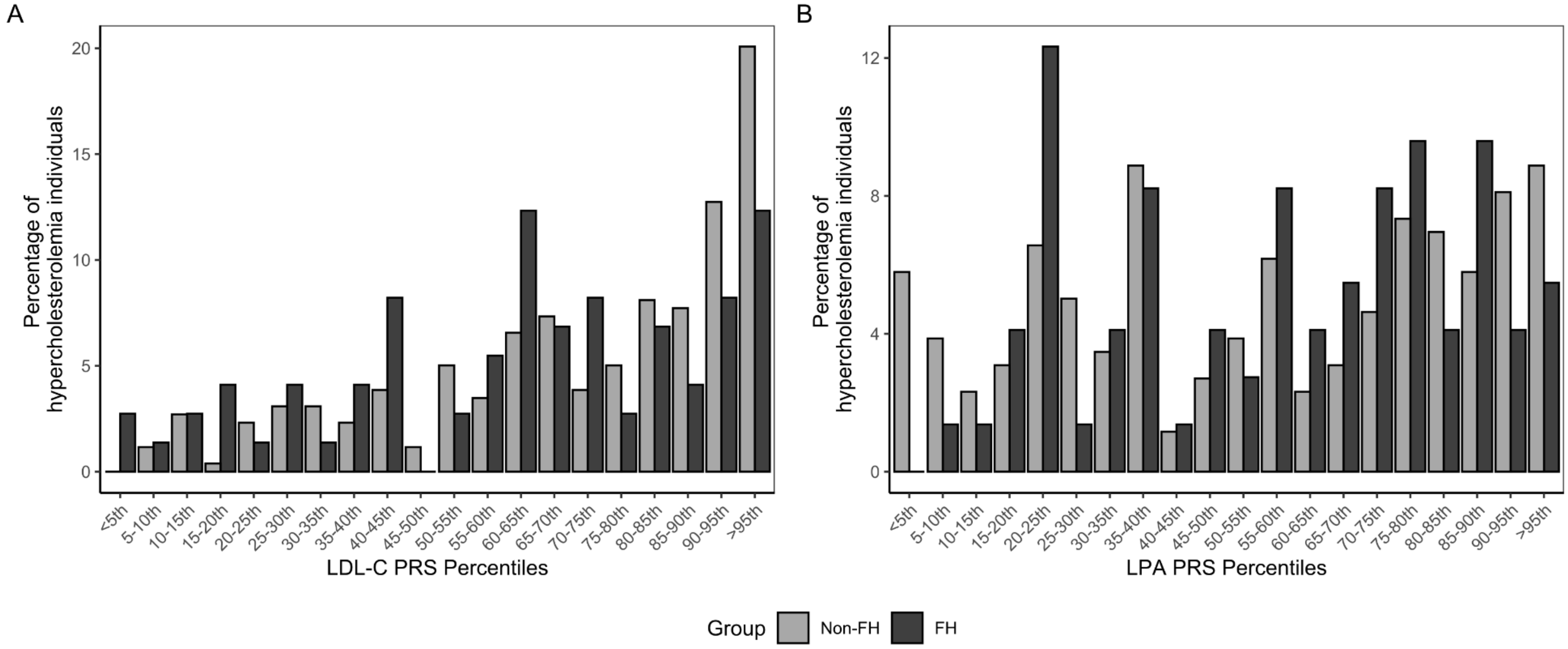

Figure S1. The percentage of FH (black bars, n = 73) and non-FH (gray bars, n =259) individuals in (A) LDL-C PRS percentiles and (B) LPA PRS percentiles relative to the respective group size.
LDL-C- low-density lipoprotein cholesterol, LPA - gene encoding lipoprotein(a); FH - familial hypercholesterolemia; non-FH – patients without P/LP variants; PRS - polygenic risk score.

Table S2. Variants of uncertain significance found in Latvian FH cohort.

Disease	Gene	Variant	rsID	ClinVar ID	Zygosity, VEP Consequence	Clinical significance	ACMG/AMP criteria	Number of cases
**FH**	***APOB***	c.10508C>T (p.Ser3503Leu)*	rs375284245	548033	Het, missense variant	VUS	PM2, PP4, PS4_Supporting, BP4	1
		c.11477C>T (p.Thr3826Met)	rs61744153	237735	Het, missense variant	VUS	PS3_Supporting, PP1, BP4	1
		c.11744C>G (p.S3915C)	rs201990496	NA	Het, missense variant	VUS	PM2, BP4, PP4	1
		c.11911G>A (p.Glu3971Lys)*	rs373477107	334088	Het, missense variant	VUS	PM2,BP4,PM3,PP4	2
		c.11952C>T (p.Thr3984=)	rs1447813723	927683	Het, synonymous variant	VUS	PM2,BP4,BP7,PP4	1
		c.12137G>A (p.Arg4046Gln)	rs149273387	334082	Het, missense variant	VUS	BP4	1
		c.13480_13482del (p.Gln4494del)*	rs562574661	265896	Het, nonframeshift deletion	VUS	PM4, PS3_Supporting	1
		c.2630C>T (p.Pro877Leu)	rs12714097	490389	Het, missense variant	VUS	.	3
		c.3670C>T (p.Arg1224Trp)	rs148959244	252482	Het, missense variant	VUS	PM2,BP4,PP4	1
		c.3743C>T (p.Thr1248Ile)	rs990797090	NA	Het, missense variant	VUS	PM2,BP4,PP4	1
		c.5124A>G (p.Leu1708=)	rs1572784788	NA	Het, synonymous variant	VUS	PM2,BP4,BP7,PP4	1
		c.6366T>C (p.Ala2122=)	NA	2953916	Het, synonymous variant	VUS	PM2,BP4,BP7,PP4	1
		c.6592A>G (p.Ile2198Val)*	rs1423439118	1895430	Het, missense variant	VUS	PM2,BP4,PM3, PP4	1
		c.6596C>T (p.Ala2199Val)*	rs1051387461	2936567	Het, missense variant	VUS	PM2, PS4_Supporting, PP4, BP4	1
		c.7285T>A (p.Ser2429Thr)	rs72653092	218448	Het, missense variant	VUS	BP4	3

Disease	Gene	Variant	rsID	ClinVar ID	Zygosity, VEP Consequence	Clinical significance	ACMG/AMP criteria	Number of cases
FH	***APOB***	c.7615G>A (p.Val2539Ile)	rs148170480	334125	Het, missense variant	VUS	BS1, PP3	4
		c.7726C>T (p.Arg2576Cys)	rs767146440	925404	Het, missense variant	VUS	BP4	1
		c.8134C>T (p.Arg2712Cys)	rs372245645	630608	Het, missense variant	VUS	PM2,BP4,PP4	1
		c.9430A>G (p.Thr3144Ala)*	rs767395919	1766945	Het, missense variant	VUS	PM1,PM2,BP4,PP4	1
	LDLR	c.829G>A (p.Glu277Lys)	rs148698650	183097	Het, missense variant	VUS	BS3	1
		c.1070A>T (p.Glu357Val)*	NA	1783245	Het, missense variant	VUS	PM2, PP3, PM5_Supporting, PP4	1
		c.1274A>T (p.Asn425Ile)	rs879254850	251762	Het, missense variant	VUS	PM2, PP4	1
		c.1503G>A (p.Ala501=)*	rs368889457	251876	Het, synonymous variant	VUS	PM2,PS4_Supporting,PP3,PP4	1
		c.1661C>T (p.Ser554Leu)*	rs879254976	251960	Het, missense variant	VUS	PM2, BP4, PS4_Supporting, PP4	1
		c.2141-9T>G*	NA	NA	Het, splice site variant	VUS	PM2,PP4,PP3, PS4_Supporting	4
		c.2324T>C (p.Val775Ala)	rs780300776	440691	Het, missense variant	VUS	PM2,BP4,PP4	1
	LDLRAP1	c.376G>A (p.Asp126Asn)	rs763971300	2146167	Het, missense variant	VUS	PM2, BP4, PS4_Supporting, PP4	1
		c.451C>T (p.Arg151Trp)	rs148916767	296979	Het, missense variant	VUS	PM2, BP4, PS4_Supporting, PP4	3
		c.488A>C (p.Gln163Pro)	NA	NA	Het, missense variant	VUS	PM2, PP3, PP4, PS4_Supporting	1
		c.850G>A (p.Ala284Thr)	rs144622500	1677169	Het, missense variant	VUS	BP4	1
	PCSK9	c.421G>A (p.Asp141Asn)	rs769000401	924431	Het, missense variant	VUS	PM2, BP4	1

Disease	Gene	Variant	rsID	ClinVar ID	Zygosity, VEP Consequence	Clinical significance	ACMG/AMP criteria	Number of cases
**FH**	***PCSK9***	c.670G>C (p.Asp224His)*	rs1644684119	1755041	Het, missense variant	VUS	PM2, PP4, BP2, PS4_Supporting	3
		c.1948G>A (p.Val650Ile)	rs767706622	369877	Het, missense variant	VUS	PM2,BP4	1
**Other**	***ABCG5***	c.121C>G (p.Leu41Val)	rs1030894919	1312062	Het, missense variant	VUS	PP4,PM2,BP4	1
		c.155G>T (p.Arg52Met)	rs758798787	NA	Het, missense variant	VUS	PM2,BP4,PP4	1
		c.505G>A (p.Glu169Lys)	rs780727528	NA	Het, missense variant	VUS	PM2, BP4	1
		c.593G>A (p.Arg198Gln)	rs141828689	284636	Het, missense variant	VUS	PP3	12
		c.656C>G (p.Pro219Arg)	rs1487073918	2959394	Het, missense variant	VUS	PM2,PP3,PP4,BP2	2
		c.1082C>G (p.Ser361Cys)	rs776365033	336044	Het, missense variant	VUS	PM2,BP4	1
		c.1327C>T (p.Pro443Ser)	rs941990198	3227756	Het, missense variant	VUS	PM2, PP4, BP2	1
	ABCG8	c.15G>T (p.Ala5=)	NA	NA	Het, synonymous variant	VUS	PM2,PP4,BP4,BP7	1
		c.636G>A (p.Gly212=)	rs770637125	NA	Het, synonymous variant	VUS	PM2,BP4,BP7,PP4	1
		c.712G>A (p.Glu238Lys)	rs34754243	291264	Het, missense variant	VUS	.	2
		c.1160C>T (p.Pro387Leu)	rs150654176	500519	Het, missense variant	VUS	BP4	1
		c.1212-7T>A	rs759300542	501909	Het, splice site variant	VUS	PM2,PP3,PP4	1
		c.1547A>G (p.Tyr516Cys)	NA	NA	Het, missense variant	VUS	PM2,PP3, PP4	2
		c.1705T>C (p.Ser569Pro)	rs776335488	2739113	Het, missense variant	VUS	PM2, BP4	1

Disease	Gene	Variant	rsID	ClinVar ID	Zygosity, VEP Consequence	Clinical significance	ACMG/AMP criteria	Number of cases
**Other**	***ABCG8***	c.1828_1830del (p.Arg611del)	rs767706636	1597858	Het, nonframeshift deletion	VUS	PM4,BP4	2
		c.1858C>A (p.Leu620Ile)	rs746858868	2893146	Het, missense variant	VUS	PM2,PP4,BP4, BP2	1
		c.1924G>A (p.Ala642Thr)	rs113005049	288467	Het, missense variant	VUS	BP4	2
	APOE	c.434G>A (p.Gly145Asp)	rs267606664	478904	Het, missense variant	VUS	PM2, PS4_Moderate	1
	LIPA	c.379C>T (p.Arg127Trp)	rs140686447	528226	Het, missense variant	VUS	PM2,BP4,PP4	1
	LPA	c.2945+1G>A	rs201480327	NA	Het, splice site variant	VUS	PP3,BP2	1
		c.3606G>A:p.Arg1202=	rs1431523386	NA	Het, synonymous variant	VUS	PM2,BP4,BP7,PP4	1
		c.3609A>C:p.Thr1203=	NA	NA	Het, synonymous variant	VUS	PM2,BP4,BP7,PP4	1
		c.3770C>T (p.Thr1257Met)	rs41267819	NA	Het, missense variant	VUS	BP4	1
		c.5543G>T (p.Gly1848Val)	rs372597108	NA	Het, missense variant	VUS	PM2, PP3, PP4	1

FH - familial hypercholesterolemia; VEP - Variant Effect Predictor; ACMG/AMP - American College of Medical Genetics and Genomics/Association for Molecular Pathology; Het – heterozygous; VUS – variant of uncertain significance; * – variant has been prioritized for segregation analysis and need one more criteria (moderate or supporting) to be promoted to “likely pathogenic”.

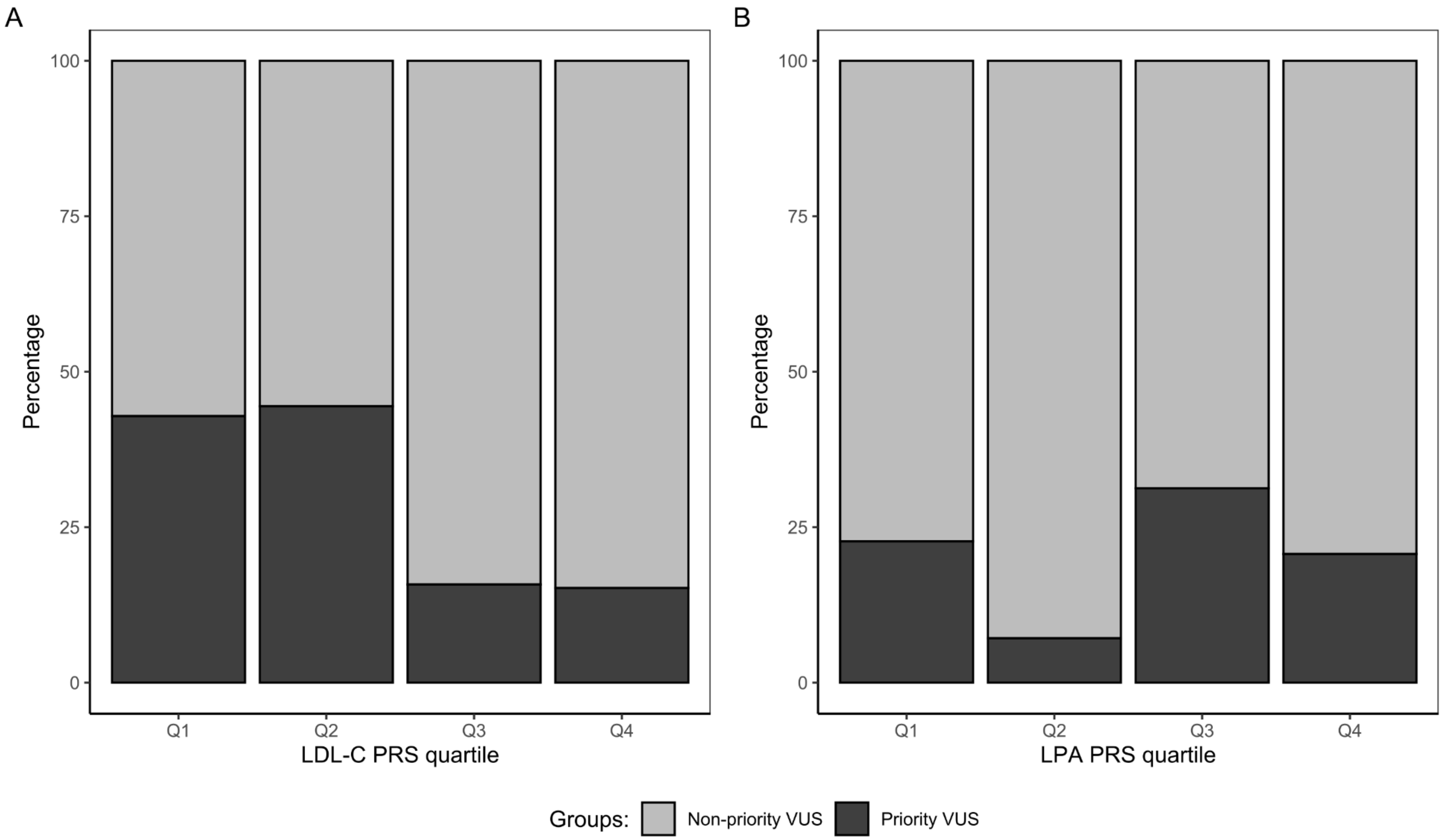

Figure S2. The proportion of priority (black bars) and non-priority (gray bars) VUS-carrying individuals in (A) LDL-C PRS quartiles and (B) LPA PRS quartiles. LDL-C - low-density lipoprotein cholesterol, LPA - gene encoding lipoprotein(a); VUS - variant of uncertain significance; PRS - polygenic risk score.
